# High-Tech Training for Birds of Prey

**DOI:** 10.3390/ani11020530

**Published:** 2021-02-18

**Authors:** Giovanni Granati, Francesca Cichella, Pia Lucidi

**Affiliations:** 1 Falcong SRLS, via Sabotino 56, 64021 Giulianova, Italy; granati83@yahoo.it (G.G.); falcong@yahoo.it (F.C.); 2 Faculty of Bioscience and Technology for Food, Agriculture and Environment, University of Teramo, via Renato Balzarini 1, 64100 Teramo, Italy

**Keywords:** birds of prey, muscle hypertrophy, endurance, rehabilitation

## Abstract

**Simple Summary:**

The stress of confinement and rehabilitation is not the only issue for a rescued wild raptor: After release, it must also deal with the new area of releasement, and hopefully, survive. This can be a true challenge for different reasons, the first of which is how long a bird has been hospitalized. High-tech rehabilitation could enhance the recovery of rescued raptors by maximizing their muscle strength, speed, and/or endurance power. The more varied the training, the more the bird will regain a muscular structure comparable to a healthy and vigorous wild raptor. Moreover, high-tech training could minimize the raptors’ attachment to humans, which may become the main problem for wild bird rescue centers, when the released raptors exhibit habituation to humans as food suppliers.

**Abstract:**

Raptors are some of the most at-risk groups of birds in the world and saving these top predators is essential for maintaining the health of many ecosystems. After hospitalization, raptors are often released when muscular recovery is still unfitting when they are unable to hunt efficiently and are at risk of dying from starvation within a few days. On the other hand, if a convalescent bird is trained with the only use of classic falconry techniques, it is likely to remain dependent on the caretaker/falconer even long after the release, so unable to hunt independently. To overcome these problems, a new training method was conceived, which could improve raptors’ muscular strength while limiting habituation to humans. This has been possible due to the combination of classic falconry techniques and modern technologies, such as the introduction of specific workouts with drones. Three falconry raptors and one wild Eurasian hobby were trained through high-tech falconry to develop the ability to catch, grasp, and airlift their prey at a different speed, altitude, and resistance. The main findings of this study were: (i) The rapid increase of the raptors’ speed; (ii) the muscular growth and endurance, and (iii) successful reintroduction of a wild bird.

## 1. Introduction

Due to the role that raptors play in their environment, they can be defined as bioindicators, demonstrating, through their presence, how healthy habitat is [1,2], thereby, improving the chances of a rehabilitated raptor to survive once released into the wild is essential. Raptors are some of the most threatened birds. Almost 18% of raptors are threatened with extinction and 52% have declining global populations [3]. Therefore, conservative life strategies must be urgently improved. In the meantime, a focus on the correct recovery of injured subjects is paramount since every single bird bears a unique, valuable, genetic code. For those species threatened with extinction, the prevision of a successful post-releasing life is an absolute requirement for the spreading of their gene into the wild population [4]. Too often, however, injured raptors, although admitted alive to rescue centers, end up euthanized [5,6,7]. Euthanasia is commonly practiced in some facilities to avoid the costly and time-consuming efforts to devote to each single rescued raptor for its treatment and maintenance [8]. For a rehabilitation center, taking care of animals belonging to a high trophic position such as raptors can be more difficult and time-consuming than other species. However, raptors’ rescue and protection are extremely desirable.

The stress of confinement and rehabilitation is not the only issue for a rescued raptor: After release, it must also cope with the new area of releasement and, hopefully, survive. This can be a true challenge for different factors, the first of which is how long a bird has been hospitalized. The longer the time of recovery, the bigger is the chance that the area (which often overlaps with that of the rescue) may have been occupied by other competitors. In most cases, a hospitalized bird is released after regaining weight and wildness. However, caretakers cannot predict the bird’s subsequent effective survival rate in the wild because this can only be verified retrospectively. As it is known, a rehabilitated predator must be able to find, capture/kill and airlift a prey that sometimes is heavier than the bird itself. For example, Gaston [9], described that an *Accipiter gentilis* can catch birds and mammals up to the size of grouses and rabbits. Therefore, physical strength recovery (not only general weight regaining) is an actual fitness prerequisite that will determine the raptor’s fate in the wild, mainly in the first few days. Csermely [10], in fact, stresses that half of the released birds of prey die from starvation within one week of release.

Rehabilitation standards and pre-release screening can be different from one rehabilitation center to another but usually, a rescued raptor is considered recovered after having regained weight, being able to fly, and completed a molt [8,10,11]. Although a short recovery period and quick release are always desirable, in case of prolonged hospitalization it could be useful to wait for a molt. This proves to be true especially when the bird’s plumage has been damaged because it could compromise the flight even in presence of a proper muscular recovery. In some cases, birds are trained by stimulating their flight in a big aviary or with the use of falconry techniques [12,13,14]. Nonetheless, these techniques have been charged with side effects: a wild bird could for example; (i) refuse to eat or inflict self-traumatism during confinement; (ii) not experience the full predatory sequence (and die for starvation in the wild), or (iii) habituate to humans [4,7,8,15,16].

This study was carried out to investigate a pioneering method of training, which combines classic falconry techniques with modern technologies. The study involved one wild and three human-raised raptors belonging to the Falconidae and Accipitridae families. This contribution aims at sharing with the scientific community the results of these combined techniques, which allows overcoming the unfitting muscular development of a raptor during hospitalization and the habituation to humans as food suppliers.

## 2. Materials and Methods

In order for a rescued raptor to hunt successfully in the wild, while avoiding habituation to humans or location, it becomes mandatory to develop a flexible training technique tailored to the subject and its predatory pattern. In the wild, when a predatory sequence is successful, a bird of prey must pick up and carry its prey, which can sometimes be heavier than the bird itself. The hunting action consists of grabbing the prey on the ground and then airlifting it, trying to get as high as possible.

In humans, it has been demonstrated that applying a weight equal to 10% of body mass can potentiate subsequent sprint acceleration performance whit an effect that may differ according to the adequate recovery [17]. Moreover, when greater forces are produced over a given period, greater acceleration is produced, resulting in a faster velocity [18]. For this reason, to strengthen the inactive musculature of the bird, and simulate the work needed to airlift real prey, we applied some weight (described further ahead) to the birds to attain a stronger musculature. We also experimented that the more the training turns out to be varied, the more the bird gains a muscular structure comparable to a healthy and vigorous wild raptor. This is true for hospitalized raptors, as well as for lazy raptors that develop vices when trained with classic falconry. This technique turns out to be particularly useful to train at their best the birds competing in Falcon races.

The training consisted of airlifting an appropriate and bearable weight according to the species, strength, and training period. The training consisted of a warm-up phase, a workout with overloads, and a period of cool down. We used this method with the raptors signaled in Table 1: three falcons (*F. subbuteo*; *F. peregrinus*; *F. peregrinus* × *F. biarmicus*) renowned for attacking prey from high altitude in a fast dive, and one Northern goshawk (*Accipiter gentilis*), with its “reckless” hunting style and its ability to attack the prey from behind and below.

During its first three years of life, the hybrid Peregrine × Lanner falcon was trained with the exclusive use of classic falconry techniques (lure). In this period, he developed some bad habits, like landing instead of flying. Therefore, together with the younger Peregrinus and Northern goshawk, its training was replaced by the new technique.

The experimental study was carried out on one wild *F. subbuteo* too. This young Eurasian hobby was introduced by some rangers who had found it near to death, skinny, and with poor chances of survival. They first forced the bird to eat some fresh quail meat and some energizer, then provided it with medications and therapy. However, due to the long period of inactivity in the aviary, the raptor gained too much weight and a patent muscular atrophy.

Raptors’ exercise regimes consisted of different steps, which varied in intensity, duration, and repetition of the workouts. The intensity changed from time to time depending on the response of each individual to the previous cycle. The duration was limited to one training session every 48 h. Each session lasted 45–60 min, which included all the different steps (from taking the bird out of its shelter, warm-up, exercise, breaks, and cool-down. According to Hall [19], we calibrated more repetitions to build strength and endurance, avoiding increasing two different factors at the same time (e.g., intensity and duration together) but rather one at a time. Together with the progress in speed, resistance, acceleration, we always monitored the raptors’ weight.

Each step was dedicated to specific purposes and consisted of different activities and workouts, adapted minute-to-minute according to the muscular and psychological response of the trained raptor. Below the description of the complete training steps, summarized in Table 2.

*Habituation* (HA) is a simplified training or pre-training. It consists of the period needed by the raptors to get used to the instruments and the activities they will perform to gain prey (for example tracking the mono-wing). This step is always present, regardless of the species. Little by little, the subject learns to follow the technological system introduced. Habituation lasts from 10 days onwards, according to the individual bird’s response. HA is a basic but pivotal phase, which can influence the following steps, for example, if the raptor should fail to associate the chosen system to the food reward. Basically, regardless of the system used (mono-wing, drone), the goal is to ensure the raptor becomes accustomed to the system, as it happens with the lure when trained through classic falconry techniques. To do this, we start with the system rolling very distant from the aviary and slowly bringing it closer, day by day, for a while. Once accustomed to the system and its noise all the training is carried out as for classical falconry. The only difference is that the food is connected to the drones and no longer with the rope. At first, the drone is moved just a little to facilitate the grasp of the food, then the bird should learn to gain the reward on its own, meter by meter until it is considered ready to follow the system flying at a certain distance. At this point, one or more of the different following regimens can be adopted for each bird.

*Explosive strength* (ES) is a step focused on the ability to express high muscular tension in the shortest time, i.e., sprint acceleration. This skill is necessary to perform rapid movements when there is a limited time to produce force or to make a quick change in direction [18,20]

*Hypertrophy* (HY) was used to gain maximum muscle growth, i.e., increasing the muscle cross-sectional area and work capacity [18,21]. HY gives the possibility to develop strength in the medium term (up to 1 km of beaten flight). This cycle is ideal for birds that fly directly on their prey, for example, Northern goshawks (*A. gentilis*). This training is strictly combined with ES because the two techniques are similar in the typology of training, although they differ in the percentage of extra load applied. In the ES cycle, where the goal is the improvement of sprint acceleration, loads are higher (75 to 95%) and the covered distance shorter (100 m), while in the HY (whose goal is the muscular growth) loads are intermediate (65 to 75%) and the distance to be covered longer (200–300 m).

*Resistance* (RE) is a training that aims at developing long-term resistance to flight by using lower loads (10 to 60%) for long distance (up to 1000 m). The lower the load, the more the training will develop long-term resistance. According to Kraemer and Ratamess [22], resistance training can increase muscular strength, local muscular endurance, and power improvement: progressive (but small acute) increases in overload are important to promote the interplay between nervous and muscular system and motor unit recruitment.

We called *Stabilization* (ST) the period of transition between two phases. Stabilization is needed for muscle recovery. Its duration depends on the extent and severity of the loss of muscular strength during hospitalization, underpinning the raptor’s compliance to the performance demanded before a new set of workouts.

Excluding habituation and stabilization, the duration of the single cycles could vary from 10 to 30 days.

To determine the weight to be applied, we developed the following Formula:

(1) $$overload=\frac{\;overload\;value\;\left(\%\right)\;\times \;bird^{\prime }s\;weight\;\left(g\right)\;}{2\times 100}.$$

Example of calculation: 10% overload for a 500-g raptor means that the weight to be airlifted by each bird’s limb should be 25 g (10 × 500/200).

All raptors were trained with the use of technological equipment such as the Berghwing (Berghwing is an automated mono-wing mini aircraft, created by the South African falconer Peter Henry Bergh of the Royal Shaheen Company, Dubai, UAE) a drone, or both combined.

The first mini aircraft weighs 965 g, with a width of 102.6 cm, a length of 60 cm, and a height of 4 cm; the propeller diameter is 6.5 cm; the maximum altitude is 500 m, and the maximum speed is 155 km/h. This new technology not only brings the twists and turns of natural flight in the training, imitating real hunting and prey escaping, but it also proved to be very agile and safer for raptors, since the engine is covered.

The drone, a modified version of the drone Drocon Bugs 3 (MJX R/C technic, Shantou, China) is a powerful mini aircraft that can fly for about 20 min thanks to a 7.4 V 1800 mAh lithium-ion battery and a recharge time of 240 min. Interestingly, the drone is equipped with a support that can be adapted to various action cameras (GoPro, C4000, or similar). Moreover, the drone can respond to commands up to 300/500 m in the open field, through a 2-way communication with a radio controller. It includes the parachute-lure, and an aerodynamic, anti-breakage, and antishock device to unhook the parachute-lure. The engine propeller is partially covered, made of soft plastic and, even in case of accidental collision, the raptor will not be subjected to severe injuries.

According to the specific step of the training, the drones have been used to replicate the natural predation, through individual exercises that reproduce the same scenarios a bird would face while hunting in nature. This means that according to the species to be trained (but also to the subject’s response) the sessions were modified, by preferring partly or for the most a high flight experience, or focus on sprint, or sudden changes of direction. For this reason, we tested different systems, preferring those with the best target performance.

The entire training has been validated by recording the raptors’ performances with the use of a GPS, the BSPlanet (Bitrabi Innovation Group, Bologna, Italy). The system, appropriate to the purpose of the study and lightweight (15 g), enables different parameters to be calculating in real-time: maximum speed, average speed, flight time, rise time, drop time, position, and altitude. In addition, each bird was weighed before, and after, each training session (usually one training session every 48 h).

The training regimens and the specific workouts of the different raptors are given in Table 3. In general, we can stress that optimal physical condition can be reached through mixed training, converging in a balanced development of the three workouts: ES, HY, and RE. This does not exclude specializing the type of workout according to:

*Species*: For sparrowhawks, for example, it could be necessary to focus on Explosive Strength, while Explosive Strength training is less suitable for eagles or buzzards that need to be trained by preferring resistance workouts; a falcon training instead, should focus on hypertrophy and resistance;

*Performance*: We must account for differences in the performance requested for recreational or memorial representations compared to what is asked to birds recruited for the Falcon races, that need a workout centered on the increase of muscular hypertrophy;

*Wild raptors*: Those birds of prey to be reintroduced in nature, regardless of the species, require an increase in their optimal fitness in the shortest time possible. The considerations made for the different species of captivity bred raptors are not always suitable for wild raptors. Prior to choosing the specific workouts, the falconer/trainer must be taken into consideration the typology and severity of the injury or illness, the stress level, wildness, species, age, weight, etc. It is for this reason that the workout of a wild raptor can be focused on resistance, hypertrophy, or explosive strength, depending on the specific circumstances, respecting the fundamental rule that is handling wild raptors as little as possible.

Therefore, the temporal progression of the training focused on constant but low overload for longer distance to attain RE development, up to intermediate or high overload for a minimal amount of time, and shorter distance for the development of HY, or ES, respectively.

In detail: the hybrid Peregrine × Lanner falcon was trained throughout all five phases, and muscle development was reached with a mixed technique based on Berghwing and drone. For the male Northern goshawk (*A. gentilis*), we chose a training made up of four microcycles (HA, ES, ST, ES), for the development of maximum horizontal speed. The training regimen used for the Peregrine falcon was a slow workout focused on chasing first the drone and then the Berghwing, to increase its resistance. The RE phase was repeated three times and was focused on reaching the maximum height. The training method used for the wild Eurasian hobby (*F. subbuteo*) was made to gain a maximum flight height together with the development of resistance. This program lasted three months, one of which was conducted during the hospitalization in the aviary. Briefly, the falcon underwent a three phases schedule (HA, RE, ST), with the habituation carried out together with 20% overloads in the aviary, followed by a period of conditioning to the drone. The second cycle consisted of two cluster sets: first, a workout focused on resistance, where the bird was trained to chase the drone with 10% overload, and the second focused on chasing the drone without overload. Each RE cycle ended with a recovery phase from 3 to 5 min.

We could not use the GPS device on the Eurasian hobby for practical reasons. In fact, because of the little weight of the species, the bird could not wear a device weighing 15 g. Anyway, the wild Eurasian hobby concluded its training after three months because at this time he demonstrated to be able to catch, grab and lift the prey at every trial. The bird was then released into the wild and monitored with a light telemetry system for more than one month.

The data has been evaluated with descriptive statistics; where possible, an analysis was performed by utilizing Wilcoxon test for non-parametric data [23].

## 3. Results

To analyze the results obtained by the hybrid Peregrine × Lanner falcon, we compared its performance with high-tech training (Berghwing) with the baseline performance when exercised through classical falconry. The results are given in Table 4.

Since the other raptors that were younger than the hybrid exercised only through the high-tech training, we can compare their performances before, and after, the workout regimens.

**Figure 1:**
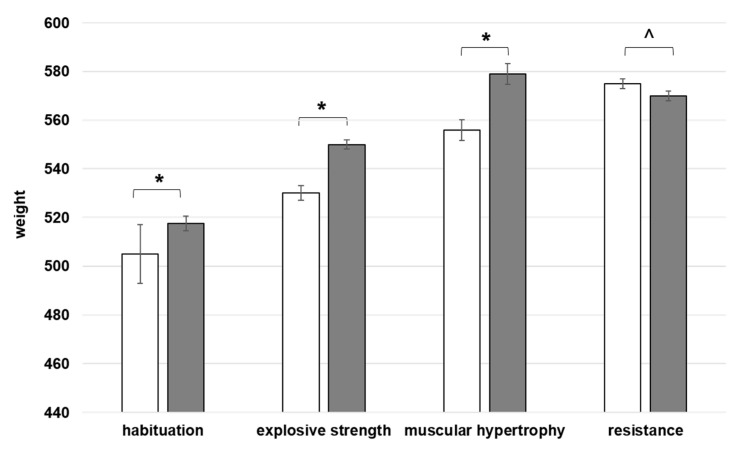
The trend of Peregrine × Lanner falcon’s weight (measured before and after each session) during the high-tech training. Each step of the training consisted of different sessions, at a distance of 48 h. Median value ± standard error of the raptor’s weight gained during the entire high-tech training, composed of four different workouts. Comparison between the results of the 1st (white bins) vs. the 2nd week (grey bins) of each regimen. Wilcoxon test for non-parametric data, results are given as a range * W = 0, *p* < 0.001; ^ W = 1.5, *p* > 0.2.

After introducing the new technologies, an increase in the hybrid Peregrine × Lanner falcon’s weight has been registered in all the training phases (W = 0, *p* < 0.001), except for the last one. During the RE program, the bird’s weight shifted indeed toward lower values, both in the first and in the second week (Figure 1).

**Figure 2:**
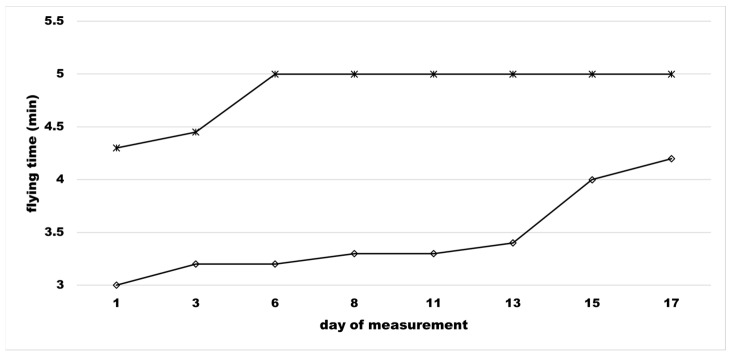
Peregrine × Lanner falcon’s flight time. Median value of the raptor’s flying time (min) during the resistance micro-cycle. At the top (∗), the value of flying time at ground level, at the bottom (◊) flying time at high altitude.

During the HY workouts, the raptor gained a higher but not significant horizontal speed in the second week (not shown). The median flying time modestly increased too, both on the ground level and in the high-altitude flight (Figure 2).

**Figure 3:**
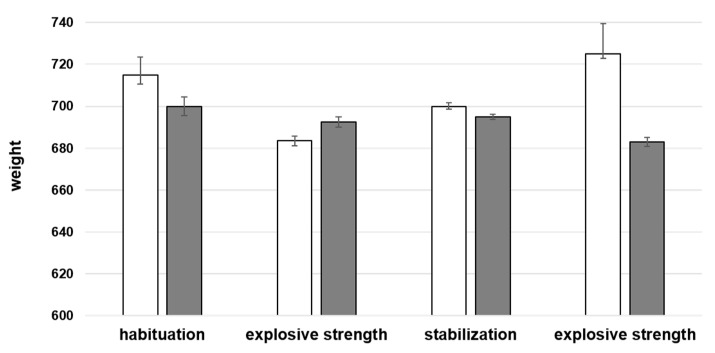
Trend of Northern goshawk’s weight during the high-tech training. Median value ± standard error of *Accipiter gentilis*’s weight gained during the entire training. White bins: 1st week; grey bins: 2nd week. Wilcoxon test for non-parametric data, results are given as a range (always W > 0, *p* > 0.2).

The Northern goshawk’s weight was always quite stable (Figure 3); the horizontal speed slightly increased (from 65 to 95 km/h) in both the ES workouts without any significant variation, while the flight distance passed from 300 to nearly 1000 m at the end of the training (not shown).

**Figure 4:**
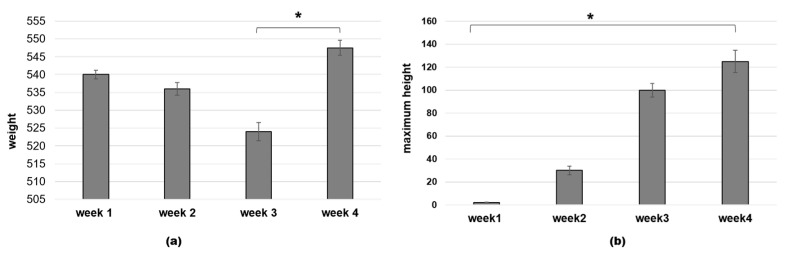
Peregrine falcon’s achievements during the habituation cycle. Median ± standard error of weight; and (**a**) maximum height (**b**). Wilcoxon test for non-parametric data, results are given as a range * W = 0, *p* < 0.001.

The Peregrine falcon’s weight declined gradually but not significantly during the general training, to significantly increase again (*p* < 0.001) during the last cycle (Figure 4a). At the same time, his maximum height increased always consistently (*p* < 0.001) during the whole training period, reaching its maximum at 400 m (continuous flapping time) (Figure 4b).

**Figure 5:**
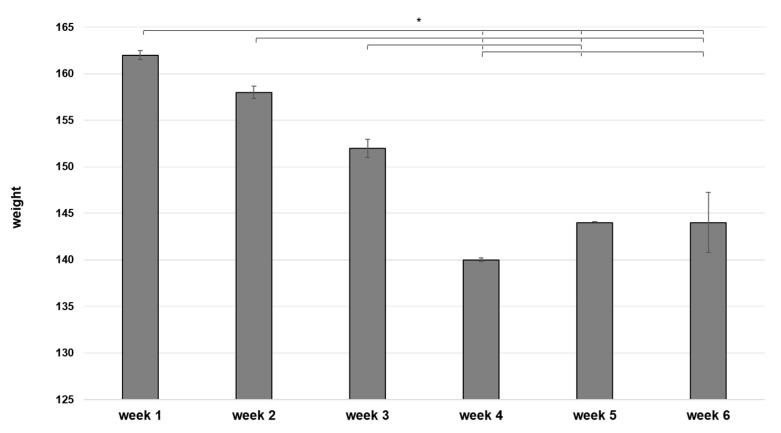
Trend of Eurasian Hobby’s weight during the high-tech training. Median value ± standard error representing the trend of the weight for the wild falcon (*F. subbuteo*) during the habituation phase. Wilcoxon test for non-parametric data, results are given as a range * W = 0, *p* < 0.001.

The wild raptor (*F. subbuteo*) had to cope with a three-month regimen consisting of different phases of habituation and resistance workouts. During this period, the weight showed an initial decline followed by a regain (Figure 5), with a final weight of 180 g and a maximum height of 100 m (continuous flapping flight) at the releasing time.

## 4. Discussion and Conclusions

The purpose of the present research was to share the results of a new high-tech training method applied to raptors. This training technique could have several advantages since it fulfills the birds’ requirement to survive autonomously in the wild, reduces the training time, avoiding too much contact with humans and also allows better predictions of the successful release of the animals. The post-traumatic recovery of a bird of prey is quite similar to that of a 100-m/marathon runner: Nobody would ever expect that a human champion would be able to compete again just because he/she regained weight or muscles flexibility. Yet, an athlete only competes to win the competition while a raptor has one essential goal to achieve after hospitalization: To survive!

The major findings of this study were: (i) the increase in speed; (ii) increase of muscular growth and resistance; and (iii) the successful reintroduction of a wild Eurasian hobby, after the verification of its ability to smoothly catch, grasp and airlift a prey.

Due to this training method, it has been possible to avoid many of the problems spotted in adapting classic falconry techniques to reintroduction. The uniqueness of this method is that it reproduces all the different situations that raptors could face once reintroduced in nature better than classic falconry. We realized that birds trained through this approach were much more responsive when chasing the prey compared to the same bird trained exclusively with classic falconry techniques (the hybrid Peregrine × Lanner falcon). This could be due, at least in part, to the fact that all these raptors easily became accustomed to the presence of drones, which maybe were not seen as a possible threat. What we have noticed throughout the study is that each bird, while chasing the drones, was much more concentrated on the prey. In this condition, it was very unlikely they could impact with the propeller, and despite our prior worries, the problem never emerged: Both the system turned out to be safe at a similar rate.

However, and while each raptor received a tailored schedule for maximizing its potentiality, some limitations have sometimes occurred. For example, in the case of the Northern goshawk, the results of the ES trials had shown that the starting point of the second cycle had returned to the level of the first cycle, meaning that muscle strength maybe should have required a longer stabilization time.

Many rehabilitation programs requiring the use of blood samples, physiotherapy, bandage, immobilization, etc. [11,12] have proven to be particularly stressful for birds of prey. Unfortunately, since quite often severely injured, ill, or malnourished raptors remain on the ground when humans approach to rescue them [8], these procedures are applied necessarily. After, the way used to regain fitness beyond veterinary medical care should be to transfer the bird to specialized caretakers that should be able to return the raptor to a psychophysical optimum to be released to the wild.

One of the underlying assumptions of raptors’ rehabilitation is that a released bird survives and resumes its “*normal*” activities [24]. For example, for a Peregrine falcon, this could mean the need to reach up to 150 km/h during horizontal flight, and more than 320 km/h when nose-diving to attack the prey [25]. In our study, the only wild rehabilitated raptor (*F. subbuteo*) was not exercised focusing on velocity, but rather on the ability to reach the maximum height.

Optimal fitness is an important result to be achieved to survive in the wild. Hunting does not always have a positive outcome for a raptor, but many attempts are needed before satisfying the birds’ efforts. A fat, weak bird, although recovered from illness or trauma, may be unable to catch for a long time and may need several attempts. Most rehabilitation processes take several months before the limbs return highly functional, which in raptors is a strong constraint to achieve a successful release [11]. When isometric resistance and stabilization exercises—to develop muscle strength and grip accuracy—are made manually, e.g., by applying some resistance to the movement of the injured limb, as in the case described by Estay-Stange and colleagues [11], there could be a high-stress risk for the hospitalized bird. Moreover, human manipulation and restraint must be carried out many times a week during the whole recovery period. Nevertheless, the rates of force development, i.e., the rate of rising in force over the change in time (also termed explosive strength) could only be gained through resistance to flight [18].

Regrettably, sometimes the opportunities to improve conservation and management outcomes have been lost as physiological studies had not fully incorporated behavioral approaches [26]. Yet, the behavior is still an effective proof of animals’ fitness. For example, monitoring the height of birds’ flight can also reflect their muscular strength; the same applies to the observation of respiratory frequency, i.e., if the bird shows no rest and the breathing is not labored, giving up the place to obvious breathing up [19].

Falconry is inscribed on the UNESCO Representative List of the Intangible Cultural Heritage of Humanity and falconers are active conservationists [13,27] and besides, experts in the behavioral ecology of the raptors they take care of. A classic falconry has been demonstrated to be a technique that can improve the raptors’ fitness more than exercise them in the aviaries [12]. However, it can be expensive and long-lasting, and at risk of birds’ habituation to humans as food suppliers. High-tech training instead, can adapt a weight to the bird without a direct manual resistance, and avoiding stress and habituation to humans too.

Fajardo and colleagues found that a released individual shows greater mortality due to starvation in the first four weeks than local wild birds [cited in 6] and can survive only overcoming the initial period of trauma/illness [28]. Therefore, the ability to achieve muscular strength enabling to fly high, and grasp prey is a cornerstone of rehabilitation. The lack of money available for many rescue centers has sometimes pressed toward a minimal hospitalization time and quicker decision making or lower threshold criteria for euthanasia [7]. Rehabilitation centers should indeed improve the survival rate, conciliating the limited resources with maximal survival and fitness success. High-tech training could have great potential in the rehabilitation of birds of prey before their release, also minimizing the time of their recovery and expenditures.

The modern technologies here summarized, could allow the development of training programs adaptable to different wild raptors to be reintroduced in nature, as long as the rescue centers can realize such a program through qualified caretakers, specialized in the use of drones, falconry techniques, and raptors’ biology.

## Figures and Tables

**Table 1:** Signaling of the four raptors trained with the new high-tech falconry.

Common English Name	Scientific Name	Sex	Age	Imprinting
Hybrid peregrine × Lanner falcon	*Falco peregrinus* (Tunstall, 1771) × *Falco biarmicus* (Temminck, 1825)	male	3 years old	parents-imprinted
Peregrine falcon	*Falco peregrinus* (Tunstall, 1771)	male	9 months	parents-imprinted
Northern goshawk	*Accipiter gentilis* (Linnaeus, 1758)	male	2 months	human-imprinted
Eurasian hobby	*Falco subbuteo* (Linnaeus, 1758)	male	≈2 months	wild

**Table 2:** Training phases for specific purposes. Workout sequence. Each raptor received a tailored exercise regimen, which could consist of two or more phases (HA being a common step for all of them).

Step	Description	Goal	Overload	Distance	Recovery Time
Habituation (HA)	Period of adaptation to the new situation	Make the raptor confident toward the device’s noise and movements	0 to 20%	-	-
Explosive strength (ES)	The combination of overload-distance and recovery that sustains and optimizes the length-tension relationship and cross-bridges formation	Gain maximum speed	75 to 95% (high)	100 m (short)	3–5 min
Hypertrophy (HY)	A step similar to ES, with decreased overload and longer fly time to underpin the muscle’s potentiation	Develop the necessary strength for medium-duration flights	65 to 75% (intermediate)	200–300 m	1 min
Resistance (RE)	A step that promotes the growth of slow-twitch fibers, and endurance activities	Develop the necessary strength for longer-duration flights	10 to 60% (low)	500–1000 m	3–5 min
Stabilization (ST)	Period of muscle recovery between two workouts	Allow the muscle to stabilize and recover from the stress accumulated during the entire training, and substitute drones with natural predation	0%	-	-

**Table 3:** The table shows the different raptors and the according workouts individually chosen for their training. Legend: n.a. = not applied.

Animal	Habituation (to)	Explosive Strength	Hypertrophy	Resistance
Peregrine × Lanner falcon	Berghwing, drone	85% constant overload	70% constant overload	high altitude(without ovedrloads)
Northern goshawk	Berghwing, drone	85% overload 25% inclination Berghwing (70 m) 90% inclination drone (30 m)	n.a.	n.a.
Peregrine falcon	Berghwing, drone	n.a.	n.a.	Step 1: 10% overload max height (drone) Step 2: free flight without overload (Berghwing)
Eurasian hobby	20% overload in aviary (after drone only)	n.a.	n.a.	Step 1: 10% overload max height (drone) Step 2: free flight without overload (drone)

**Table 4:** Performances of Peregrine × Lanner falcon trained by classic or high-tech falconry. Flight time and speed (not available for manual technique) reached by the raptor and registered every ten days from the 10th to the 40th day of training.

Item	Day 10	Day 20	Day 30	Day 40	Technique
Flight time (min)	01:10	01:43	02:02	02:30	Classical falconry
	01:33	01:59	02:28	02:52	Berghwing
Speed (km/h)	--	--	--	--	Classical falconry
	68	75	84	93	Berghwing

## Data Availability

Authors confirm that all relevant data generated during this study are included in the article.
